# Supplementary figures and images for: Predictive Factors for Decreasing Left Ventricular Ejection Fraction and Progression to the Dilated Phase of Hypertrophic Cardiomyopathy
Source: J Clin Med. 2023 Aug 5;12(15):5137. doi: 10.3390/jcm12155137 (PMC10420074; doi:10.3390/jcm12155137)

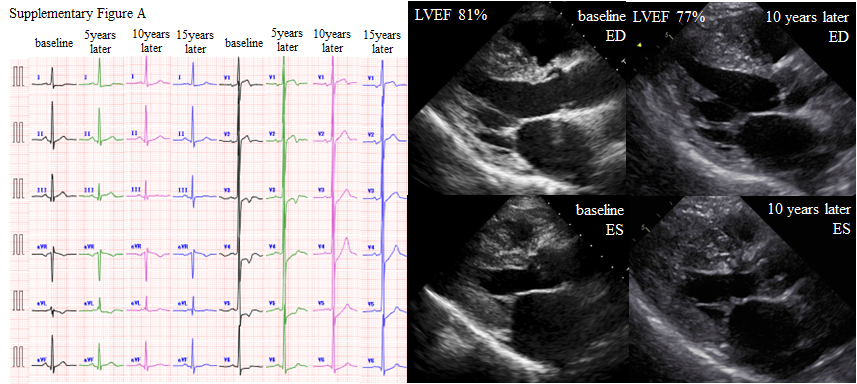

Supplement: Supplementary file 1 [file jcm-12-05137-s001.zip › Supplementary Figure S1.tif]

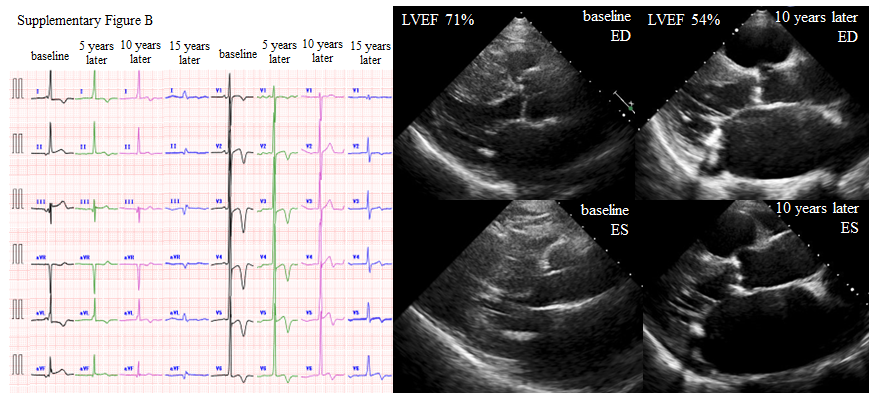

Supplement: Supplementary file 1 [file jcm-12-05137-s001.zip › Supplementary Figure S2.tif]

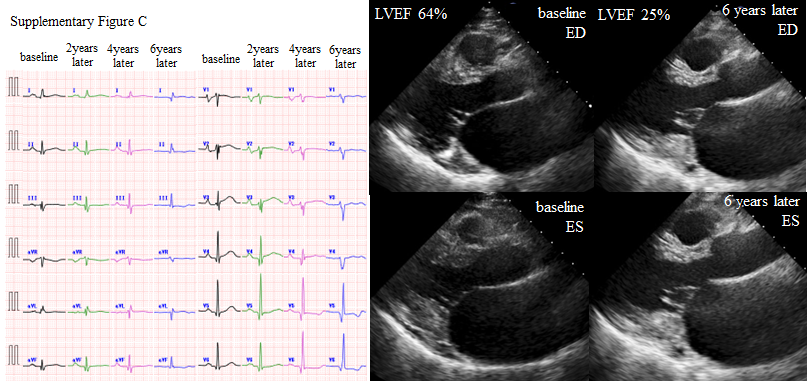

Supplement: Supplementary file 1 [file jcm-12-05137-s001.zip › Supplementary Figure S3.tif]
